# Impact of climate on the population dynamics of an alpine ungulate: a long-term study of the Tatra chamois *Rupicapra rupicapra tatrica*

**DOI:** 10.1007/s00484-018-1619-y

**Published:** 2018-10-02

**Authors:** Michał Ciach, Łukasz Pęksa

**Affiliations:** 1 Department of Forest Biodiversity, Institute of Forest Ecology and Silviculture, Faculty of Forestry, University of Agriculture, al. 29 Listopada 46, 31-425 Kraków, Poland; 2 Tatra National Park, ul. Kuźnice 1, 34-500 Zakopane, Poland

**Keywords:** Mammal, Ruminant, Herbivory, Climate change, Mountain, Protected area

## Abstract

Global warming is considered as a phenomenon having a negative effect on animals living in cold climate. However, herbivorous species inhabiting cold zones may potentially benefit from increase of temperature as this influence duration of vegetation period and increase food resources. In this study, we analyze the impact of climate factors on the long-term dynamics of an isolated and unhunted population of the Tatra chamois *Rupicapra rupicapra tatrica*. The population growth rate, based on autumnal chamois counting carried out from 1957 to 2016, were correlated with a set of climatic variables. We tested the hypothesis that high temperatures in summer could have a positive impact on the population, since they influence vegetation growth, which ensure food resources. On the other hand, heavy falls of snow and long-lasting and deep snow cover could adversely affect the population by reducing population survival during the winter. The results of this study indicate that climatic variables best explaining the autoregressed population growth rate (from the autumn of year *t*-1 to the autumn of year *t*) were the mean summer temperature of year *t*-1: the population increase was greater following a warmer summer in year *t*-1 and, in lower extent, the total precipitation during winter: the population decrease was greater following a winter with heavy snowfall. Duration and thickness of snow cover have no negative effect on population growth rate. The results indicate that the population dynamic of the Tatra chamois is determined in the long term by weather conditions, mainly by temperature, when kids are birthing and growing. The results of this work highlight that climatic changes may be responsible for the population dynamic of high-mountain species. Climate warming may lead to increase in duration of vegetation period in cold climatic zones, what may in turn have positive effect on herbivorous species, which relay upon food resources limited by low temperatures within vegetation period.

**Electronic supplementary material:**

The online version of this article (10.1007/s00484-018-1619-y) contains supplementary material, which is available to authorized users.

## Introduction

During the last 100 years, the mean global temperature has risen by about 0.6 °C and this has had a significant effect on the phenology of plant flowering, the functioning of populations, and the migration of animals (Root et al. 2003; Karl and Trenberth 2003). Current scenarios predict a further increase in temperature, which may lead to the synergism of rapid temperature rise and other stresses, in particular, habitat destruction. Species associated with high mountain environments and the Arctic region are most at risk in the context of these forecast climate changes (Beniston et al. 1997; Lemoine et al. 2007). The fauna of mountains is associated with specific vegetation zones, and the presence of these is determined by climatic conditions. Ongoing global warming will presumably cause the elevation of the upper limits of vegetation layers to rise, in consequence leading to the disappearance of species associated with specific zonal habitat conditions (Beniston et al. 1997; Thomas et al. 2004).

The impact of climate changes on a global scale is preceded by local or regional reactions of species to changes in weather parameters (Stenseth et al. 2002). These local reactions of animals and plants make it possible to predict global trends (Walther et al. 2002), which often manifest themselves only after a considerable lapse of time and have a marked influence on the survival of a population in the longer time perspective (Gaillard et al. 1997; Post et al. 1997). Climate changes can affect whole population, but surveys exploring the extensive ranges of species are difficult to implement in view of populational differentiation, migrations, or the extremely diverse kinds of pressure on environments. This is why the consequences of the long-term impact of abiotic environmental factors on the dynamics of whole populations of species are still poorly understood. This applies as well to the populations of adapted to cold climate animals living in extreme environmental conditions such as those of high mountain regions.

Apart from impacting on habitats, climate can have a direct influence on animals, altering their physiological, behavioral, and ecological reactions. Temperature is the most significant abiotic factor governing the life processes of organisms (Krebs 2009), affecting reproduction, survival, and growth rate (Sæther 1997). At the same time, temperature fluctuations alter the duration and thickness of snow and ice cover, which has important consequences for the animal species relying on its presence (Moline et al. 2008). Such a situation is especially likely in Arctic or mountainous regions, which are particularly sensitive to climatic changes, and where there is a high risk of ecological endangerment in the form of progressive changes to the natural habitats of cryophilic species (Parish and Funnell 1999; Watson and Haeberli 2004). In polar regions, even minimal changes in temperature can cause serious environmental perturbations (Curran et al. 2003), and in the case of highly specialized, stenotopic species like polar bears *Ursus maritimus* or ringed seals *Phoca hispida* may lead to their extinction (Stirling et al. 1999; Derocher et al. 2004; Ferguson et al. 2005).

Alpine species, like Alpine chamois *Rupicapra rupicapra rupicapra* or species inhabiting mountain environments, like red deer *Cervus elaphus*, although adapted to cold climate, depend on weather conditions, which is often manifested by the population drop in the year following winter with extremely low temperatures and high precipitation (Willisch et al. 2013; Bonardi et al. 2017). However, reaction of populations inhabiting mountainous regions on climate changes during growing season is not well documented. As in the case of Arctic species, changing climatic conditions can affect reproduction and mortality in a population, and a high temperature can have a greater impact on cryophilic species than a low one. However, global warming may have a particularly strongly influence on vegetation phenology in cold climate and lead to increase in duration of vegetation period (Kullman 2004). In consequence, this may have a positive effect on herbivorous species inhabiting cold climatic zones, which heavily relay upon food resources limited by low temperatures within vegetation period.

The objective of the present work was to determine the long-term impact of weather factors on the dynamics of a population of high-mountain animals adapted to cold climate using the example of the isolated and unhunted population of the Tatra chamois *Rupicapra rupicapra tatrica*. This population is isolated from other regions of species occurrence and does not undertake vertical migration but spends the entire year in the open terrain above the tree line, an area that is under strict national park protection (Jamrozy et al. 2007). We tested the hypothesis that the year-to-year population growth rate of the Tatra chamois is governed in the long term by climatic conditions. We assumed that heavy falls of snow and long-lasting and deep snow cover could adversely affect the population. However, high temperatures in summer could have a positive impact on the population, since they influence vegetation growth, which ensure food resources during chamois’ kids birthing and growing.

## Methods

### Study area

Lying in the central part of the western Carpathians, the Tatra Mountains are the highest mountain massif between the Alps and the Caucasus (the height difference is 1755 m, and the tallest peak is Gerlach 2655 m amsl). Covering an area of some 800 km^2^, the Tatras extend latitudinally over a distance of nearly 60 km and are about 20-km wide. The Tatras are built of granitoids, metamorphic, and limestone rocks. The area has a distinctive post-glacial relief with an altitudinal zonation of climate and vegetation (lower montane, upper montane, subalpine, alpine, and sub-nival zones). The Tatras are protected in their entirety in the form of national parks—the Tatranský Národný Park (TANAP) in Slovakia (formed in 1949), which cover ca. 80% of the massif, and the Tatrzański Park Narodowy (TPN) in Poland (formed in 1954). In addition, the Tatras have been declared a Man and Biosphere Reserve and included in the Natura 2000 network of protected areas in Europe. Due to strict conservation regime, hunting is not allowed and poaching is not recorded (Jamrozy et al. 2007).

The natural environment of the Tatras is shaped largely by the climate, the wide diversity of which is the upshot of inflowing air masses of different geographical origin (Limanówka et al. 2008). In the alpine zone of the massif, the mean annual temperature is − 0.7 °C, and the coldest month is February. There are, on average, 188 winter days, when the mean daily temperature (*T*_mean_) is < 0 °C, whereas a thermal summer (when *T*_mean_ > 15 °C) does not occur at all. The growing season (when *T*_mean_ > 5 °C) lasts for just under 100 days. Snow covers the ground for an average of 221 days in the year, usually from December to April, but snow may sometimes persist well into May and June, hindering access to foraging areas and exacerbating the risk of triggering avalanches. The snow layer is no more than 50-cm-thick only from mid-June to early September. Snow may occur sporadically in July and August: during these months, the mean daily temperature is > 7.5 °C. The mean annual precipitation is nearly 1800 mm. The thermal conditions prevailing in the Tatras, expressed as air temperature, resemble those of the Alps (Niedźwiedź 2006).

### Tatra chamois data

For this analysis, we used the results of the annual chamois counting carried out from 1957 to 2016 by TPN and TANAP, obtained from the databases of both institutions. These counting are done in November (exceptionally somewhat later if the weather conditions so dictate). The counting methodology is based on the one suggested back in 1932 by J. Müller (Chudík 1969). The entire area of the Tatras inhabited by chamois was divided into counting areas, each of which was patrolled during 2 days by a team of at least two people. Population of Tatra chamois does not show vertical movement over the year and occupy open habitats located above the tree line, exclusively (Jamrozy et al. 2007). The counting areas were so distributed as to enable coverage of the entire terrain; there were up to 30 in TPN and up to 60 in TANAP. Only animals actually sighted were counted; tracks or other indirect signs of the presence of chamois were ignored. The numbers recorded by individual observers were checked by the coordinators from both parks in order to prevent the multiple counting of the same herds by different observers. The total number of animals counted was taken to represent the overall number of animals in the population. The fieldwork was carried out by qualified personnel from both, Polish and Slovakian Tatra national parks. For organizational reasons or if the weather was too poor, counts were not undertaken in 1979, 1981, 1985, 1987, and 1997. For these years, the numbers of chamois were estimated by the national park authorities based on the annual observations and records collected throughout the year by the park rangers. Information on age and sex composition was not collected annually and/or for all study regions, making them unsuitable for analyses.

Counts of the whole population are used frequently in open areas to assess population size (Morellet et al. 2007). In favorable conditions (rain- and fog-free weather and cloud-base height above summits) and with a large number of observers densely covering surveyed area, this used method assumes over 90% of the total population to be counted (Chudík 1969). However, true detection probability of applied counting methodology and, therefore, accuracy of method, have not been tested at the time of its implementation and throughout the entire monitoring period. Since the number of animals in a given year could be underestimated when reported based on counting, this result should be treated as a minimum population size.

### Climatic data

For the purposes of this paper, we used meteorological data from unpublished reports kindly made available to us by the Institute for Meteorology and Water Management (a state research institute), characterizing the weather conditions prevailing in the different months from 1956 to 2016. The database contained information for each month on the mean air temperature, total precipitation, maximum snow cover thickness, and numbers of days with snow cover. These figures came from the high-mountain weather observatory on the Kasprowy Wierch (located at 1987 m amsl), which lies in the central part of the Tatra massif, in the zone inhabited by chamois.

### Data handling and analyses

The number of chamois (*N*) in a given year (*t*) was used to present long-term population trend. Based on the number of the chamois (*N*) in years *t* and *t*-1 population growth rate (*λ*) was calculated (Sibly et al. 2003; Turchin 2003) with the following formula:

$$ \lambda ={N}_t/{N}_{t-1} $$

Following Ferreira et al. (2016), we modeled population growth rate in a given year (*λ*_t_) as a function of past population sizes in current (*t*) and previous (*t*-1 … *t*-*i*) years. The linear autoregressive model was:

$$ {\lambda}_t={\upalpha}_t\times {N}_t+{\upalpha}_{t-1}\times {N}_{t-1}+\cdots +{\upalpha}_{t-i}\times {N}_{t-i}+{\upvarepsilon}_t $$

where α_*t*_ … α_*t*-*i*_ are the slopes of the relationship between λ_*t*_ and population size in a given year (*t* … *t*-*i*) and represent the strength of density dependence, and ε_*t*_ represents the process noise. The density dependent model set included a total of seven models starting from the global model with population sizes with time lags from 1 to 5 years (*i* = 5). Then, successive models had their last time lag (1 year) removed progressively, until null model (intercept only) was received. Competing models were compared with the Akaike’s information criterion (Burnham and Anderson 2002) corrected for small samples (AICc). The resulting models were subsequently ranked in order of increasing AICc. The differences between the models with the lowest AICc were calculated (ΔAICc) for each of the resulting models.

The best supporting model of population growth rate contain two variables: the number of the chamois in years *t* and *t*-1 population (Table 1). The figure obtained, taken to be a dependent variable, expressed the autoregressed population change from the autumn of year *t*-1 to the autumn of year *t*. Calculation of annual population growth rate based on the counting results (see data source) could be biased by error in estimation of population size in a given year, what may lead to increase of variance. Due to lack of detailed knowledge on accuracy of applied counting method, we were not able to improve counting results and have to rely on calculated population growth rates.

**Table 1:** Autoregressive models for density dependent population growth rate (*λ*_*t*_) of Tatra chamois *Rupicapra rupicapra tatrica* in 1957–2016: *N*_*t*_ = number of chamois in a year *t*, *N*_*t-i*_ = time lag = number of chamois in a year *t*-*i* (*i* = 1 ... 5 years earlier). Number of parameters in the model (*K*), Akaike’s information criterion for small samples (AICc), difference between best supporting model (∆AICc), and Akaike weight (*w*) are provided for each competing model

Model	*K*	AICc	∆AICc	*w*
*λ*_*t*_ = *N*_*t*_ + *N*_*t*-1_	4	− 172.3	0.0	0.933
*λ*_t_ = *N*_*t*_ + *N*_*t*-1_ + *N*_*t*-2_	5	− 167.0	5.3	0.064
*λ*_t_ = *N*_*t*_ + *N*_*t*-1_ + *N*_*t*-2_ + *N*_*t*-3_	6	− 160.8	11.5	0.003
*λ*_*t*_ = *N*_*t*_ + *N*_*t*-1_ + *N*_*t*-2_ + *N*_*t*-3_ + *N*_*t*-4_	7	− 154.9	17.5	0.000
*λ*_*t*_ = *N*_*t*_ + *N*_*t*-1_ + *N*_*t*-2_ + *N*_*t*-3_ + *N*_*t*-4_ + *N*_*t*-5_	8	− 149.5	22.8	0.000
*λ*_*t*_ = 1	2	− 58.6	113.7	0.000
*λ*_*t*_ = *N*_*t*_	3	− 58.3	114.0	0.000

A set of 12 climatic variables (Table 2) was calculated from the 48 meteorological variables (mean air temperature, total precipitation, maximum snow cover thickness, and total number of days with snow cover for each month). They related to the current year (*t*), from which the autumnal population numbers were taken, and to the previous year (*t*-1). The choice of variables characterized the weather conditions in the various periods in the Tatra chamois’ life cycle and was made on the basis of the species’ biological and ecological features (Jamrozy et al. 2007). Mating period of Tatra chamois took place from September to November (TEMP IX-XI_t-1_; PRECIP IX-XI_t-1_), when males start to approach the herds of females and display courtship behavior. This is the period when chases and skirmishes between rival males are regular occurrences. Halfway through this period, there may be heavy falls of snow, giving rise to snow cover duration (SNOWDAY X_t-1_-IX_t_) and thickness, which may persist until the spring or summer of the following year (SNOWTHICK X_t-1_-IX_t_). In March–April (TEMP III-IV_t_; PRECIP III-IV_t_), females forage intensively: they are in the final stages of their pregnancies and maintaining them requires high levels of energy. The Tatra chamois give birth to kids in May or early June. The growth of the kids and the adaptation to their environment, involving their gradual weaning from a diet of milk to a vegetarian one and acquiring the skills for moving around in high-mountain areas, take place from May to August (TEMP V-VIII_t_; TEMP V-VIII_t-1_; PRECIP V-VIII_t_; PRECIP V-VIII_t-1_). Winter, with its persistent total snow cover and lowest temperatures, covers the period from December to February (TEMP XII_t-1_-II_t_; PRECIP XII_t-1_-II_t_). The predictors within the TEMP or PRECIP groups included in the model selection process were assumed to be independent. However, to avoid between-group co-linearity, the multicollinearity of the climatic parameters was checked with Spearman’s correlation (the correlation coefficient was < 0.5 for all variable pairs).

**Table 2:** Descriptive statistics of climatic variables in the Tatra Mountains (based on the data collected through the study period 1957–2016)

Variables	Description	Mean	SD	Min	Max
TEMP IX-XI_t-1_	Mean temperature from September to November of year *t*-1	0.6	1.3	− 2.3	3.4
TEMP XII_t-1_-II_t_	Mean temperature from December of year *t*-1 to February of year *t*	− 7.6	1.5	− 11.8	− 4.3
TEMP III-IV_t_	Mean temperature from March to April of year *t*	− 4.4	1.4	− 8.4	− 1.1
TEMP V-VIII_t_	Mean temperature from May to August of year *t*	6.0	1.1	3.3	8.6
TEMP V-VIII_t-1_	Mean temperature from May to August of year *t*-1	5.9	1.1	3.3	8.6
PRECIP IX-XI_t-1_	Total precipitation from September to November of year *t*-1	360.7	124.0	133.0	748.4
PRECIP XII_t-1_-II_t_	Total precipitation from December of year *t*-1 to February of year *t*	350.6	103.6	140.3	642.6
PRECIP III-IV_t_	Total precipitation from March to April of year *t*	251.1	86.8	109.7	453.5
PRECIP V-VIII_t_	Total precipitation from May to August of year *t*	797.2	206.3	415.7	1398.8
PRECIP V-VIII_t-1_	Total precipitation from May to August of year *t*-1	792.7	207.3	415.7	1398.8
SNOWTHICK X_t-1_-IX_t_	Maximum thickness of snow cover from October of year *t*-1 to September of year *t*	197.6	64.0	85.0	355.0
SNOWDAY X_t-1_-IX_t_	Total number of days from October of year *t*-1 to September of year *t* with snow cover	219.3	18.3	186.0	276.0

Akaike’s information criterion corrected for small samples (AICc) was used for model selection (Burnham and Anderson 2002). The resulting models were subsequently ranked in order of increasing AICc. Differences between the models with the lowest AICc were calculated (ΔAICc) for each of the resulting models. Model likelihoods were normalized according to Akaike weights (*w*) to illustrate the weight of evidence of each model. In order to illustrate direction of variable effect of the most important climatic variables explaining the population growth rate of the Tatra chamois we used multivariate regression. Estimates (±SE) and 95% confidence intervals (CI) were presented. A set of all climatic variables was used as the starting model, then the backward selection procedure was run until a model was obtained in which all of the variables analyzed were significant.

Population number (growth rate) in a given year may be dependent on the population size (growth rate) in the previous year(s). Therefore, numbers of Tatra chamois and population growth rate were tested to detect autocorrelations using PAST software (Hammer et al. 2001). A distance-weighted least squares smoothing procedure was applied to illustrate long-term population dynamic of Tatra chamois and long-term trend of population growth rate (*λ*). In this smoothing procedure, the influence of individual points decreases with the horizontal distance from the respective points on the curve. The statistical procedures were performed using Statistica 12.0 software (StatSoft 2014).

## Results

### Long-term population dynamics

**Fig. 1:**
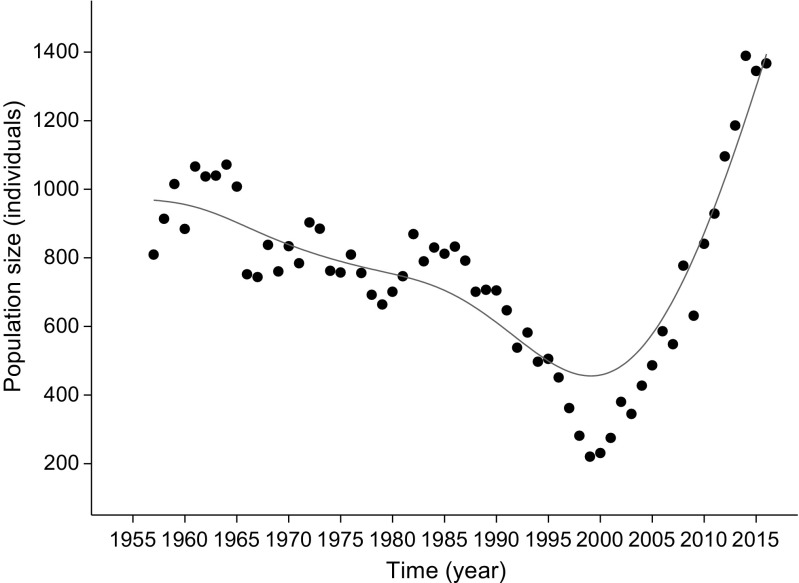
Long-term population dynamics of the Tatra chamois *Rupicapra rupicapra tatrica* in 1957–2016 (dots = total number of individuals in a given year, line = population trend smoothed with distance-weighted least squares smoothing procedure)

**Fig. 2:**
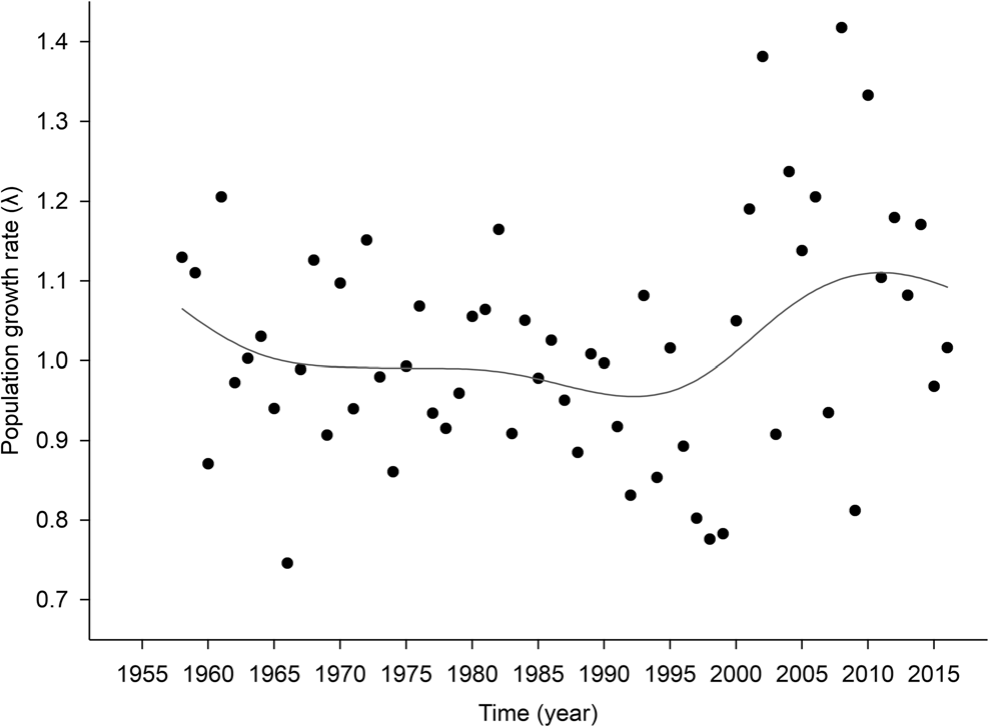
Long-term trend of population growth rate (*λ*) of the Tatra chamois *Rupicapra rupicapra tatrica* in 1957–2016 (dots = values of population growth rate in a given year, line = population growth rate smoothed with distance-weighted least squares smoothing procedure)

Between 1957 and 1999, the population of the Tatra chamois decreased from around 1100 animals in the early 1960s to 220 by the turn of the century (Fig. 1). From 2000 to 2016, however, the population increased rapidly, with numbers exceeding the level prior to the decline. The number of Tatra chamois in a given year was highly correlated with number of animals in previous years (Supplementary data – Fig. 1). From the start of Tatra chamois counts until the turn of the century, despite high variation, the long-term trend of population growth rate was stable (Fig. 2). Since 2000, however, population growth rate have been increasing long-term. The population growth rates of Tatra chamois in a given year was not correlated with population growth rates in previous years (Supplementary data – Fig. 2).

### The impact of climatic variables

**Fig. 3:**
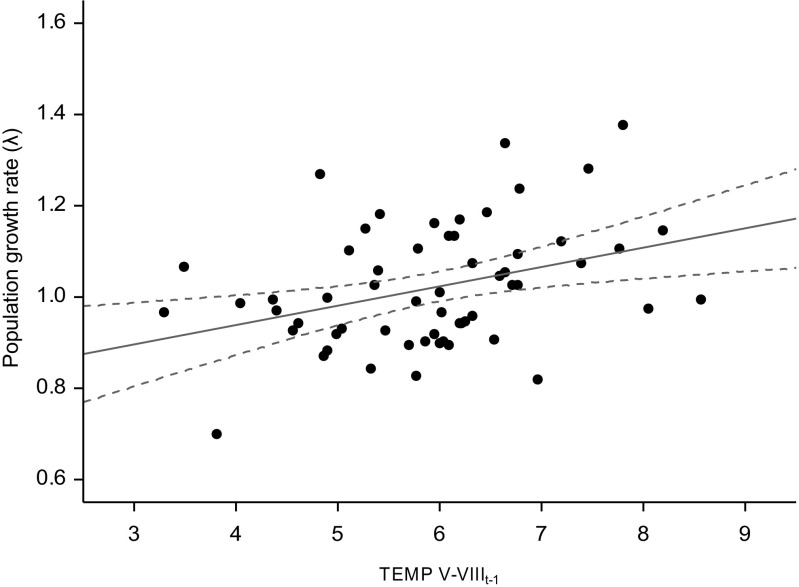
Relationship between the population growth rate (*λ*) of the Tatra chamois *Rupicapra rupicapra tatrica* and the mean temperature in the summer of year *t*-1 (TEMP V-VIII_t-1_) in 1957–2016

Based on the AICc, the model best explaining the population growth rates (Table 3) covered the mean summer temperature in the summer of year *t*-1 (TEMP V-VIII_t-1_) and the total precipitation in the winter of year *t*-1/*t* (PRECIP XII_t-1_-II_t_). Next consecutive substantially supporting model (ΔAICc < 2) included additional climatic variable: the total precipitation in the spring of year *t* (PRECIP III-IV_t_). Less substantially supporting model (ΔAICc < 5) included the maximum thickness of the snow cover persisting from the autumn of the previous year to the time when the chamois were counted (SNOWTHICK X_t-1_-IX_t_) (Table 3). The variables best explaining the population growth rates (variables with highest probabilities of being included in the best approximating models) were the mean temperature in the summer of year *t*-1 (TEMP V-VIII_t-1_) and the total precipitation in the winter of year *t*-1/*t* (PRECIP XII_t-1_-II_t_): AICc weight 0.621 and 0.271, respectively (Table 4). Multivariate regression revealed that the mean temperature in the summer of year *t*-1 (TEMP V-VIII_t-1_) and the total precipitation in the winter of year *t*-1/*t* (PRECIP XII_t-1_-II_t_) were significantly correlated with population growth rate, which was larger after a warmer summer in year *t*-1 and lower after a winter with heavy snowfall (Table 5, Fig. 3).

**Table 3:** Sets of candidate models explaining the population growth rate (*λ*) of Tatra chamois *Rupicapra rupicapra tatrica* in 1957–2016 (for variables, see Table 1 and “Methods”). Number of parameters in the model (*K*), Akaike’s information criterion for small samples (AICc), the difference between the given model and the most parsimonious model (ΔAICc), and the Akaike weight (*w*) are reported for each model

Model	*K*	AICc	ΔAICc	*w*
TEMP V-VIII_t-1_ + PRECIP XII_t-1_-II_t_	4	− 74.8	0.0	0.402
TEMP V-VIII_t-1_ + PRECIP XII_t-1_-II_t_ + PRECIP III-IV_t_	5	− 73.8	1.0	0.239
TEMP V-VIII_t-1_	3	− 72.9	1.9	0.155
TEMP V-VIII_t-1_ + PRECIP XII_t-1_-II_t_ + PRECIP III-IV_t_ + SNOWTHICK X_t-1_-IX_t_	6	− 71.5	3.3	0.077
PRECIP XII_t-1_-II_t_	3	− 71.3	3.6	0.068
PRECIP III-IV_t_	3	− 67.5	7.3	0.010
SNOWTHICK X_t-1_-IX_t_	3	− 67.4	7.4	0.010
NULL	2	− 67.2	7.6	0.009
TEMP V-VIII_t_	3	− 66.7	8.1	0.007
TEMP III-IV_t_	3	− 65.6	9.2	0.004
PRECIP V-VIII_t-1_	3	− 65.6	9.3	0.004
PRECIP V-VIII_t_	3	− 65.4	9.4	0.004
PRECIP IX-XI_t-1_	3	− 65.0	9.8	0.003
TEMP IX-XI_t-1_	3	− 64.8	10.0	0.003
TEMP XII_t-1_-II_t_	3	− 64.7	10.1	0.003
SNOWDAY X_t-1_-IX_t_	3	− 64.7	10.1	0.003

**Table 4:** AICc weights for climatic variables used in the model selection procedure explaining the population growth rate (*λ*) of Tatra chamois *Rupicapra rupicapra tatrica* in 1957–2016 (see Table 2). Values are the probabilities that a given variable is in the best approximating models. Variables ranked with decreasing AICc weights

Variable	AICc weights
TEMP V-VIII_t-1_	0.621
PRECIP XII_t-1_-II_t_	0.271
PRECIP III-IV_t_	0.031
SNOWTHICK X_t-1_-IX_t_	0.020
TEMP V-VIII_t_	0.007
TEMP III-IV_t_	0.004
PRECIP V-VIII_t-1_	0.004
PRECIP V-VIII_t_	0.004
PRECIP IX-XI_t-1_	0.003
TEMP IX-XI_t-1_	0.003
TEMP XII_t-1_-II_t_	0.003
SNOWDAY X_t-1_-IX_t_	0.003

**Table 5:** Estimates (±SE) and 95% confidence limits (CL) of climatic variables correlated with the population growth rate (λ) of Tatra chamois *Rupicapra rupicapra tatrica* in 1957–2016

Variable	Starting model							Final model						
	Effect size	±SE	Wald’s stat	− 95% CL		+ 95% CL	*p*	Effect size	± SE	Wald’s stat	− 95% CL		+ 95% CL	*p*
Intercept	− 0.4007	0.3604	1.24	− 1.1070	to	0.3056	0.266	− 0.0759	0.1107	0.47	− 0.2929	to	0.1411	0.493
TEMP IX-XI_t-1_	0.0111	0.0176	0.39	− 0.0235	to	0.0456	0.530							
TEMP XII_t-1_-II_t_	− 0.0097	0.0122	0.63	− 0.0337	to	0.0143	0.427							
TEMP III-IV_t_	0.0064	0.0125	0.26	− 0.0181	to	0.0308	0.611							
TEMP V-VIII_t_	0.0115	0.0206	0.31	− 0.0288	to	0.0519	0.575							
TEMP V-VIII_t-1_	0.0261	0.0183	2.04	− 0.0097	to	0.0620	0.153	0.0348	0.0140	6.21	0.0074	to	0.0622	0.013
PRECIP IX-XI_t-1_	0.0000	0.0002	0.00	− 0.0003	to	0.0003	0.954							
PRECIP XII_t-1_-II_t_	− 0.0003	0.0002	3.15	− 0.0007	to	0.0000	0.076	− 0.0003	0.0002	4.29	− 0.0006	to	0.0000	0.038
PRECIP III-IV_t_	− 0.0001	0.0002	0.15	− 0.0006	to	0.0004	0.698							
PRECIP V-VIII_t_	0.0000	0.0001	0.10	− 0.0001	to	0.0002	0.750							
PRECIP V-VIII_t-1_	0.0000	0.0001	0.10	− 0.0002	to	0.0002	0.746							
SNOWTHICK X_t-1_-IX_t_	− 0.0002	0.0003	0.55	− 0.0008	to	0.0004	0.459							
SNOWDAY X_t-1_-IX_t_	0.0015	0.0011	1.68	−0.0008	to	0.0037	0.195

## Discussion

The results of this work indicate that changes in the numbers of Tatra chamois are correlated with summer temperature and point to a delayed population growth, which depend on the climatic conditions of summer in the previous year. The energy requirements of females peak from late winter to mid-summer, when their metabolic rate can double during the first months after giving birth (Oftedal 1985; Robbins 1993). Therefore, in mountain ungulates, a short birth season is synchronized with forage productivity (Côté and Festa-Bianchet 2001). Moreover, ungulate population growth depends heavily on adult female survival and recent studies revealed persistent individual differences in female reproductive potential, with a positive correlation of reproductive success over consecutive years (Hamel et al. 2010; Rughetti et al. 2017). Since increase temperature of summer influence vegetation growth and dynamic (Kullman 2004), we suppose that warm seasons allow for effective rebuilt of body reserves of females after pregnancy and increase their body condition and survival. This may, in turn, influence maternal care, which directly affect offspring survival translating directly into baby mass and condition (Théoret-Gosselin et al. 2015). This lead to conclusion that higher kids survival after a warm summer is a key factor which is responsible for delayed population growth. Since winter is the crucial period for offspring survival (Willisch et al. 2013), good body condition of offspring achieved after warm summer (due to maternal care and rich summer food resources) will apparently increase their winter survival what is manifested by the population number in the following year.

In high mountain environments, as in the Arctic, the rate of metabolism in ungulates is higher than in areas with a milder climate (Hudson and Christopherson 1985; Hudson and Haigh 2002). In the cold climate, where a large amount and variety of plant food is available only during the short growing season, the metabolic rate has to be faster so as to guarantee the synthesis of tissues and to build up the body’s reserves of fat (Lawler and White 2003). This allows herbivores to reduce their daily digestible energy maintenance requirements during extended periods of energetic restriction (Strickland et al. 2005). Moreover, it has been observed that Arctic ungulates tend to adapt their physiological and behavioral mechanisms regarding their food requirements to the nutritional value of the habitat, which is probably dictated by the need to save energy (Parker et al. 1993; Hudson and Haigh 2002). However, chamois may apparently benefit from extended vegetation period, which reduce their need for energy saving.

Climate changes may have a negative effect on the condition of ungulates inhabiting the Arctic and high mountain regions (Post et al. 2003; Pettorelli et al. 2007; Post and Forchhammer 2008). Long-persisting snow cover is propitious to the winter survival prospects of adults and to fetal development in pregnant females (Post and Stenseth 1999; Forchhammer et al. 2001; Patterson and Power 2002), whereas changes in rainfall and temperature regimes could alter the length of the growing season (Sabine et al. 2002; Pettorelli et al. 2005). However, there are some concerns that is probable that the growing season will be out of step with the period when herbivores have their maximum food requirements (Post and Forchhammer 2008), leading to physio-phenological disjunction. The negative reaction to difficult weather conditions occurs in spring (Portier et al. 1998), when food availability is restricted; a delayed start to the growing season affects the opportunities for foraging, what may increase winter losses. Results of our work indicate the existence of negative relationship between population growth rate of Tatra chamois and winter precipitation.

Extreme weather conditions can increase energy outlays, depending on the age and sex structure of the population, and can govern the population numbers (Coulson et al. 2000) and sizes of herds (Fritz and Loison 2006). Precipitation was shown to have a deleterious effect and can limits the populations of ungulate (Dailey and Hobbs 1989; Chovancová and Gömöry 2000). The adverse effect of prolonged, cold winters on chamois populations was demonstrated in the Alps, for example (Willisch et al. 2013). The high altitude favored the accumulation of snow from early autumn to late spring, and the low temperatures helped to maintain the snow cover, making it difficult for chamois to replenish energy resources dissipated during the rut, in pregnancy, and before giving birth. Our study indicate that, in the Tatra chamois, total precipitation during winter have marginal but significant influence on the population. Negative effect of snow conditions was earlier pointed by Chovancová and Gömöry (2000). However, weakly pronounced influence of weather conditions during winter on chamois is probably because the snow cover is not so thick in the Tatras (they are much lower than the Alps) and potentially negative weather events are less extreme. Moreover, variable weather conditions (precipitation, wind, temperature) can also affect the choice of habitat occupied by male and female ungulates (Conradt et al. 2000), which may actively look for places with milder climate or thinner snow cover (e.g., places at former snow slides).

A significant factor limiting the expansion of the ungulate population is inclement weather at the time the kids are being born and in the first weeks of their lives. Heavy rains with concomitant drops in temperature can have a deleterious effect (Picton 1984). After a winter with thick snow, some ungulates give birth to smaller and weaker young (Adams 2005), what may have delayed negative effect on population abundance. This cumulative effect of greater energy outlays while giving birth only makes itself felt in later winters and may cause the numbers of animals in a population to fall (Patterson and Power 2002). Moreover, the thickness and hardness of the snow cover may have a potentially significant effect on how predators function. Among the Tatra predators, it is the Eurasian lynx *Lynx lynx* that is of the greatest importance, as it is the sole large predator in these mountains (the others are the brown bear *Ursus arctos* and the wolf *Canis lupus*) whose distribution range coincides with the Tatra chamois habitats (Chovancová and Gömöry 2000). The chamois may be the second-most important prey item in the lynx’s diet after the roe deer *Capreolus capreolus* (Molinari-Jobin et al. 2002); in the Tatras, the lynx is known to kill up to 20% of all Tatra chamois found dead (Jamrozy et al. 2007).

The population of herbivores depends on the availability of habitat and spatial and temporal distribution of food resources. The general climate warming may lead to expansion of woody plants, and the elevated tree line may reduce the potential habitat area of alpine ungulates (Didion et al. 2011). At present, potential chamois habitat in the Tatras covers an area of 270 km^2^ (Jamrozy et al. 2007). However, analyses of aerial photographs taken between 1955 and 2004 do not indicate the existence of significant changes in tree line in Tatras (Guzik 2008).

Our results have some limitations that should be taken into account when interpreting the results. First, the climatic conditions should be considered as one of the numerous factors, which have influence on the population dynamic. Number of individuals in a given population depend on intra- and interspecific relationships, and factors like predation, parasites, diseases, food quality and abundance, and human disturbance (Pęksa and Ciach 2018) have influence on reproduction and survival. Results of this study might be also affected by the accuracy of applied counting methodology. Due to weather conditions, number and qualifications of observers detection probability may not be constant in every year, what, in turn may have impact on number of recorded animals and lead to increase of variation.

In summary, this work has shown that the numbers of the isolated and unhunted population of the Tatra chamois, inhabiting a strictly protected national park area, is correlated with the climatic variables of a high-mountain environment. It indicates that the long-term dynamics of populations of herbivorous species living in a cold climate may benefit from increase of summer temperature as this influence vegetation growth and dynamic, which potentially increase food resources.

## Electronic supplementary material

ESM 1 (DOCX 45 kb)
