# Supplementary material for: Impact of climate on the population dynamics of an alpine ungulate: a long-term study of the Tatra chamois Rupicapra rupicapra tatrica
Source: Int J Biometeorol. 2018 Oct 2;62(12):2173–82. doi: 10.1007/s00484-018-1619-y (PMC6244863; doi:10.1007/s00484-018-1619-y)
Supplement: Supplementary file 1 — (DOCX 45 kb) [file 484_2018_1619_MOESM1_ESM.docx]

**Supplementary data**


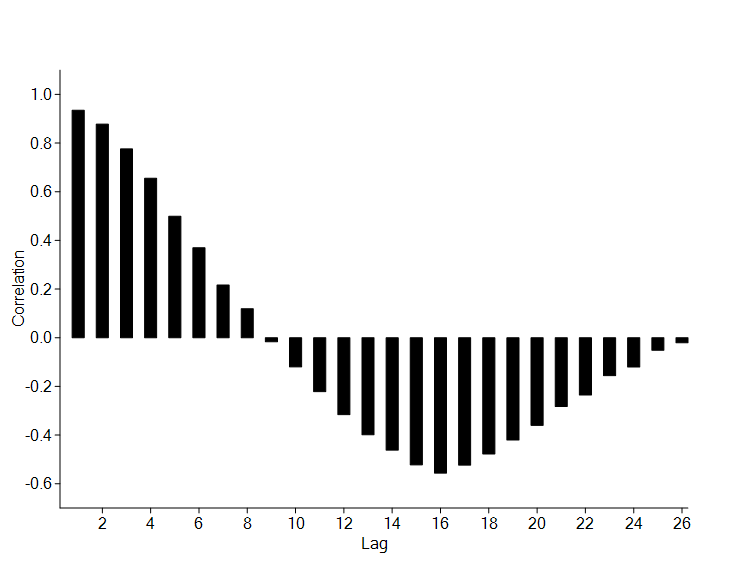


Fig. 1. Autocorrelation between the numbers of Tatra chamois *Rupicapra rupicapra tatrica*.


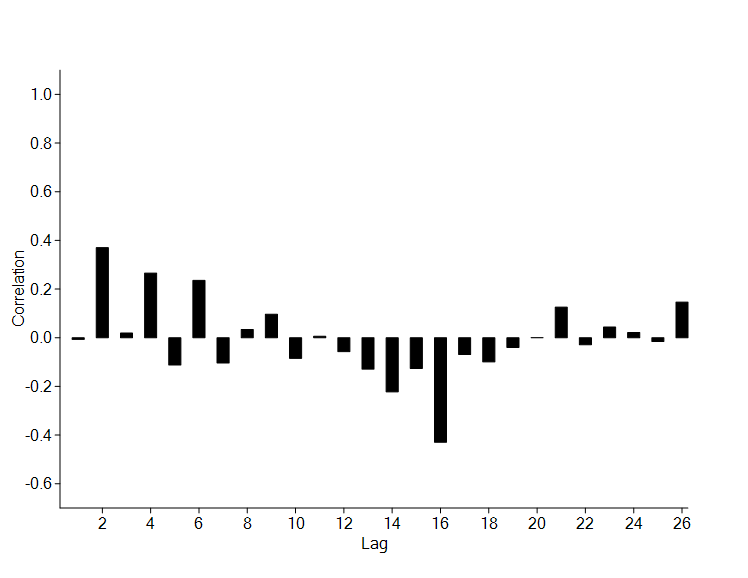


Fig. 2. Autocorrelation between the population growth rates of Tatra chamois *Rupicapra rupicapra tatrica*.
